# WHOLE GENOME TARGETED ENRICHMENT AND SEQUENCING OF HUMAN-INFECTING *CRYPTOSPORIDIUM* spp.

**DOI:** 10.21203/rs.3.rs-4294842/v1

**Published:** 2024-05-17

**Authors:** NJ Bayona-Vásquez, AH Sullivan, MS Beaudry, A Khan, RP Baptista, KN Petersen, MIU Bhuiyan, B Brunelle, G Robinson, RM Chalmers, EVC Alves-Ferreira, ME Grigg, JC Kissinger, TC Glenn

**Affiliations:** 1 Division of Natural Science and Mathematics, Oxford College of Emory University, Oxford, GA, 30054, USA; 2 Department of Environmental Health Science, College of Public Health, University of Georgia, Athens, GA, 30602, USA; 3 Institute of Bioinformatics, University of Georgia, Athens, GA, 30602, USA; 4 Daicel Arbor Biosciences, Ann Arbor, MI, 48103, USA; 5 Animal Parasitic Disease Laboratory, Agricultural Research Service, US Department of Agriculture, Beltsville, MD, 20705, USA; 6 Infectious Diseases, Houston Methodist Research Institute, Houston, TX, 77030, USA; 7 Odum School of Ecology, University of Georgia, University of Georgia, Athens, GA, 30602, USA; 8 Cryptosporidium Reference Unit, Public Health Wales, Microbiology and Health Protection, Singleton Hospital, Swansea SA2 8QA, UK; 9 Swansea University Medical School, Singleton Park, Swansea, SA2 8PP, UK; 10 Laboratory of Parasitic Diseases, National Institutes of Allergy and Infectious Diseases, National Institutes of Health, Bethesda, MD, 20892, USA; 11 Department of Genetics, University of Georgia, Athens, GA, 30602, USA; 12 Center for Tropical and Emerging Global Diseases, University of Georgia, Athens, GA, 30602, USA

## Abstract

*Cryptosporidium* spp. are protozoan parasites that cause severe illness in vulnerable human populations. Obtaining pure *Cryptosporidium* DNA from clinical and environmental samples is challenging because the oocysts shed in contaminated feces are limited in quantity, difficult to purify efficiently, may derive from multiple species, and yield limited DNA (<40 fg/oocyst). Here, we develop and validate a set of 100,000 RNA baits (CryptoCap_100k) based on six human-infecting *Cryptosporidium* spp. (*C. cuniculus*, *C. hominis*, *C. meleagridis*, *C. parvum*, *C. tyzzeri*, and *C. viatorum*) to enrich *Cryptosporidium* spp. DNA from a wide array of samples. We demonstrate that CryptoCap_100k increases the percentage of reads mapping to target *Cryptosporidium* references in a wide variety of scenarios, increasing the depth and breadth of genome coverage, facilitating increased accuracy of detecting and analyzing species within a given sample, while simultaneously decreasing costs, thereby opening new opportunities to understand the complex biology of these important pathogens.

## Introduction

Cryptosporidiosis, a disease characterized by mild to severe gastrointestinal symptoms, is caused by protist parasites in the genus *Cryptosporidium*. Infection can damage the intestinal epithelium, leading to adverse health outcomes in vulnerable human populations (young, old, immunocompromised, and pregnant)^1–3^. Transmission of *Cryptosporidium* spp. to humans may occur through direct or indirect transmission^1,4–7^. An estimated 7.5M global cases of cryptosporidiosis occur in children aged 0–24 months, and 133,422 deaths occur annually^8–10^. In the United States, *Cryptosporidium* is the third leading cause of zoonotic gastroenteritis and the leading cause of waterborne disease outbreaks^11^. In low-middle income countries, *Cryptosporidium* spp. is the second most common pathogen in infants from 0–11 months^12^. In children under five years, infection with *Cryptosporidium* spp. is the fourth leading cause of diarrheal death globally^3,12,13^. Furthermore, cryptosporidiosis is one of the original AIDS-defining illnesses and remains associated with high mortality in immunocompromised individuals^14–16^. Thus, cryptosporidiosis is globally important, affecting millions annually.

There are at least 38 recognized species within the genus *Cryptosporidium*^17^ with a wide range of host specificity and likelihood of infecting humans. Thus, *Cryptosporidium* spp. vary in public health importance^17^. At the genome sequence level, based on available sequences, there is a cluster of species (called the high-similarity group) that share greater than 80% identity across much of their genome^5,18–20^, which includes *C. cuniculus*, *C. hominis*, *C. meleagridis*, *C. parvum*, *C. tyzzeri*, and *C. viatorum*. Host-derived and environmental samples can also contain more than one species or genotype of *Cryptosporidium* spp.^6,21–25^. Mixed infections may be limited to specific geographic areas, particularly in endemic regions^26^. Most reported mixed infections contain *C. parvum* and *C. hominis*^21,23,27^. Thus, methods to detect and characterize multiple species and mixed infections are needed to understand cryptosporidiosis better.

Several molecular methods have been developed to gain insight into the genetic diversity of *Cryptosporidium* species, often relying on single marker typing of conserved (small subunit rRNA, SSU) or polymorphic regions (glycoprotein gene 60, *gp60*, or heat shock protein 70, *hsp70*)^15,28^. Other approaches for genetic characterization include multilocus sequence typing (MLST)^29–32^, and more recently, whole genome sequencing. The disadvantages of MLST include the possible unreliability of markers in different regions due to the limited information about the genetic variability of populations within *Cryptosporidium* species^32^, and thus, assays are not universal. Challenges with comprehensive genomic assays such as whole genome sequencing are constrained by difficulty in purifying oocysts^33^ and the limited amount of DNA from purified samples and from fecal clinical samples (which also have an abundance of other organisms’ DNA). These challenges have translated into the need for laborious purification methods combined with costly deep sequencing^34,35^ and the risk of failing to detect low-abundance species within mixed infection samples^28^. Therefore, methods that can characterize *Cryptosporidium* genomes from limited amounts of DNA from pure oocysts or fecal samples that can be implemented easily and reliably would be beneficial.

Hybridization sequence capture (a.k.a., capture enrichment sequencing) has well-known advantages^36–38^ relevant to studying *Cryptosporidium* spp. and other parasites: it is feasible to design thousands of baits to capture entire genomes (e.g., 9 Mb genome of *Cryptosporidium* spp.); baits can tolerate many mismatches (thus species with < 20% sequence divergence may be captured); baits can account for varying levels of sequence divergence (thus conserved regions can anchor comparisons of related taxa); and successful enrichment reduces the amount of sequencing needed (thereby reducing sequencing costs). These strengths are particularly important when samples are highly variable or poorly understood. The first *Cryptosporidium* whole genome bait set (CryptoCap_73k^39^) was designed using the *C. parvum* IOWAII incomplete genome assembly^40^, and is reported to perform well when each sample is enriched individually. However, library pools that contain diverse species showed bias during enrichment for *C. parvum* over other distantly related species^39^. Because fecal samples can contain unknown or mixed *Cryptosporidium* species, a second-generation bait set is needed to enrich genomes for a broader spectrum of *Cryptosporidium* species and consider highly polymorphic genomic regions. Enriching pools of sample libraries is also desired for time- and cost-efficiency.

To ameliorate the current challenges in the genomic study of human-infecting *Cryptosporidium* spp., we developed, validated, and critically assessed CryptoCap_100k and compared its performance relative to current molecular methods (i.e., *gp60*, *18S rRNA*, and CryptoCap_73k) for the accurate identification of *Cryptosporidium* species. CryptoCap_100k was designed from the most complete publicly available genome sequences (Table 1). We validated the sensitivity and specificity of CryptoCap_100k *in silico* and *in vitro* with simulated and mock samples, multiple bait dilutions, and library preparation methods. Then, we tested the reliability of these methods with real clinical samples. We demonstrate that CryptoCap_100k is an efficient and economical method that aids in characterizing *Cryptosporidium* genome(s) in DNA extracted from pure or fecal samples. The increased representation facilitates intra-sample genetic variation analyses and improves species identification accuracy, opening new opportunities to understand the complex biology of these important pathogens.

## Results

### CryptoCap_100k Bait Design & Characteristics

We designed a set of 100,000 120-mer RNA baits targeting six *Cryptosporidium* species (Table 1). The *C. parvum* reference sequence required 73,672 baits for full coverage. An additional 4,447 baits were needed to cover the most divergent regions of the three most similar genome sequences (*C. hominis, C. cuniculus, and C. tyzzeri*). Many more baits were needed to cover *C. meleagridis* and *C. viatorum*, and with relaxed matching criteria, we included only a subset of sequences identified for *C. viatorum*. Finally, we added baits for genes of particular interest (130 baits). The bait set is available for commercial purchase (Design ID: D10006Crypto, Cat# #308508.V5, #308548.V5, and #308596.V5, Arbor Biosciences, MI, USA).

To characterize this bait set, we mapped the baits to each of twelve whole-genome sequences from ten *Cryptosporidium* species available to date (Table 2, Supplementary Notes #1, Data Availability). Mean depth and breadth of coverage were high for the six species used for bait design (1.0X and 90.46%, respectively). These measures were much lower for four distantly related species not considered in bait design (0.08X and 7.33%; Table 2; Fig. S1). When comparing the multi-species design (CryptoCap_100k) to the single-species design (CryptoCap_73k) (Supplementary Notes #2), although the mean percent of baits mapped to the twelve genomes was slightly higher for CryptoCap_73k than CryptoCap_100k (64.73% vs. 55.6% baits, respectively), our bait set showed slightly higher mean depth and breadth across all genomes (Table 2). However, these differences were not statistically significant (p-values > 0.05) (Figs. S2-S3).

### In Silico Simulations of Enrichment and Sequencing

After simulating library construction, enrichment, and sequencing with our bait set for each of the twelve genome sequences and for two fragment sizes (Supplementary Notes #3), we observed up to 99.9% breadth of coverage with an average depth of coverage of 7.6X among all genomes (Table 3, Fig. S4). Both measures tend to increase with fragment size in all species except *C. cuniculus*. Plots of breadth and depth of coverage for those genomes with chromosome-level assemblies (*C. hominis* UdeA01 and *C. parvum* IOWA-ATCC) corroborated the continuity of simulated reads from large fragments across all chromosomes (Figs. S5- S6).

**Figure 1.**
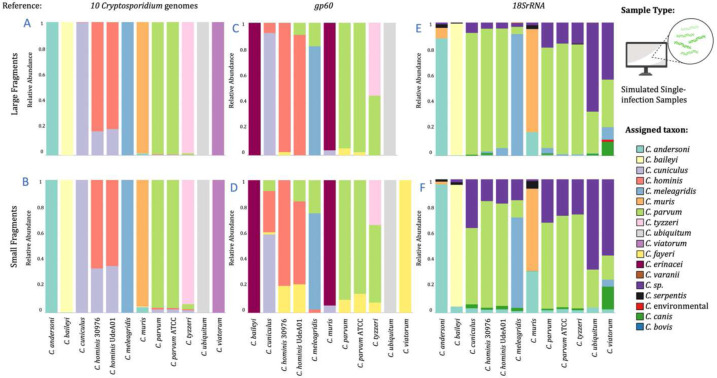
Barplots of relative abundance of species assignment from mapping *in silico* simulated enriched and sequenced reads for each of the twelve *Cryptosporidium* genomes to three reference databases. Simulations were modeled separately for two fragment-insert size classes, one of large-520 bp fragments (above) and one of small-200 bp fragments (below). Three reference databases were used to map simulated reads, one containing ten *Cryptosporidium* genome sequences (10-CrypGS), a *gp60* database (Khan et al., 2018), and an *18S rRNA* database (Xiao et al., 2010).

When simulated reads were mapped to the reference database containing ten genomes from ten different *Cryptosporidium* species (10-CrypGS), at least 99.1% of reads for each sample mapped to the corresponding genome, allowing high accuracy for species assignment for all *Cryptosporidium* species tested, including those not considered in the bait design (Supplementary Notes #4, Table S1). Differences in species assignment between short and large fragments were largely unimportant, except for *C. hominis* (Fig. 1A and 1B). However, species assignment was impacted when mapped to specific gene databases instead (Figs. 1C–F; Tables S2, Supplementary Results).

Only 894 of our baits (0.01%) map to 111 genome sequences from the 1,008 genomes database^41^, including the human genome (Supplementary Notes #5). In total, 84.2% of these baits mapped to eukaryotic, 13.9% to bacteria, and 1.9% to archaea (Fig. S7; Table S4). Most of the baits mapped with the lowest score (682 MAPQ == 0), and only 75 baits had mapping scores above 20 against 31 non-*Cryptosporidium* organisms (Fig. S8-S9).

**Figure 2.**
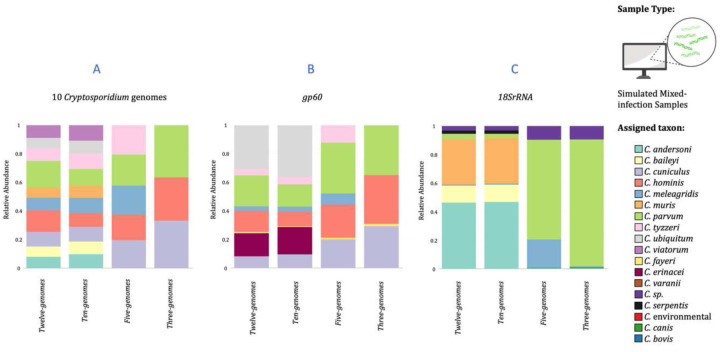
Barplots of relative abundance of species assignment from mapping four classes of *in silico* simulated enriched and sequenced mixed-infection samples. Three reference databases were used to map mixed infection simulated reads, one containing ten *Cryptosporidium* genome sequences (10-CrypGS), a *gp60* database (Khan et al., 2018), and an *18S rRNA* database (Xiao et al., 2010).

Simulated mixed *Cryptosporidium* infections (Supplementary Notes #7) showed a high proportion of reads (≧99.88%) mapped to 10-CrypGS (Table S5). All species in a sample were correctly identified for all datasets, with only slight deviations from expected relative abundances (Fig. 2A, Table S6). In comparison, mapping mixed reads to *gp60* or *18S rRNA* provided poorer proper species assignments (Figs. 2B, 2C; Tables S7-S10; Supplementary Results).

### In Vitro Simulation of Pure Infections and Mixed Infections

To further validate our bait set, the DNA of *C. parvum* and *C. meleagridis* were independently used for library construction, enrichment, and sequencing (Supplementary Notes #8; Table S11). A higher proportion of filtered reads mapped to the respective genome sequences in enriched than unenriched libraries (95.8% vs. 68.1%, p-value = 0.094; Fig. S12), reaching a fold change between 1.11X and 1.35X. A similar breadth of coverage (85.2% vs. 81.6%, p-value = 0.75) and greater depth (17.3X vs. 5X, p-value = 0.01) were observed between enriched and unenriched, respectively (Fig. S13-S14). Mapping reads to 10-CrypGS showed that for both library types, a very low proportion of *C. parvum* reads were assigned to two other species (*C. cuniculus* and *C. hominis)*; these incorrect assignments were only slightly elevated in the unenriched samples (Fig. S15; Table S12).

**Figure 3.**
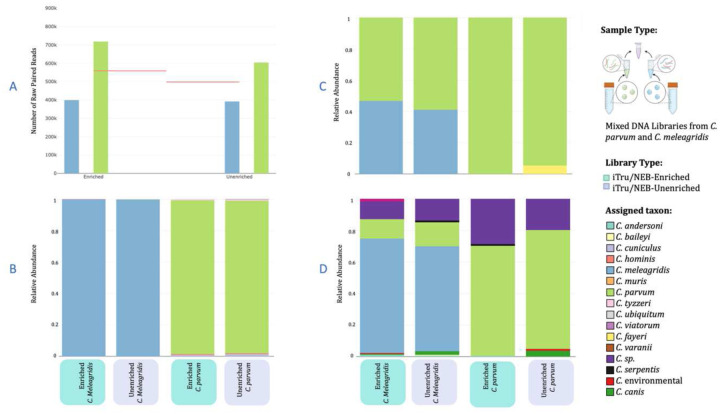
Species assignment plots from mapping one mock mixed-infection sample for which we sequenced unenriched and enriched libraries. (A) The number of raw paired reads obtained for each species in the mix, *C. parvum* in green and *C. meleagridis* in blue for both library types. The pink lines indicate the expected number of reads for each sample for a 50–50 proportion. Relative abundance of species assigned from mapping the sequence reads to (B) the ten-genome sequence reference database (10-CrypGS), (C) the *gp60* database, and (D) the *18S rRNA* database.

Then, individually indexed libraries from pure *C. parvum* and *C. meleagridis* DNA samples were pooled to mock a mixed infection (Supplementary Notes #9; Table S13). The reads from the unenriched mock sample showed that the mixture was unequal initially (60.6% *C. parvum* and 39.4% *C. meleagridis*; Fig. 3A) and that enrichment increased the bias slightly (64.3% *C. parvum* and 35.7% *C. meleagridis*; Fig. 3A; Table S13).

Mapping reads from the mock mixed sample to the three databases performed similarly to the *in silico* simulations (Fig. 3B–D; Supplementary Results). The 10-CrypGS yielded 95.7% retained reads for enriched samples and 81.4% for unenriched (p-value = 0.50). The percentages of reads that were properly assigned were very high (97% unenriched, 98% enriched for *C. parvum*; and > 98% for unenriched and enriched for *C. meleagridis*; Fig. 3B). The reads mapped to the *gp60* database performed well for the *C. parvum* sample, but less than half of the reads were correctly assigned for the *C. meleagridis* sample (Fig. 3C; Supplementary Results). Most reads mapped to the correct species in the *18S rRNA* database, but percentages were substantially misassigned (Fig. 3D; Supplementary Results).

### LOD: In Vitro Sensitivity Test with Different Inputs of C. parvum DNA

**Figure 4.**
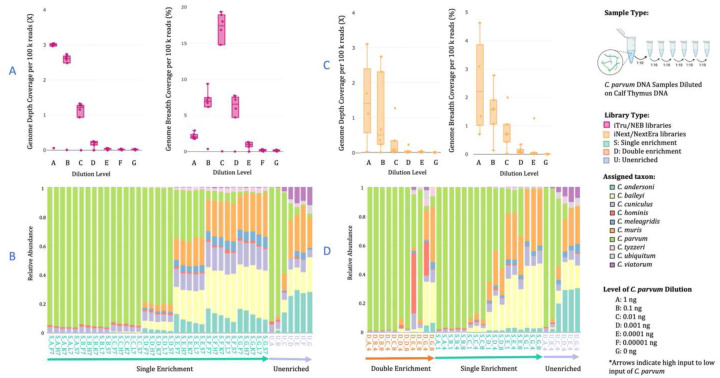
Summary statistics for sequence data from serially diluted *C. parvum* samples to assess detection levels given different input amounts. (A) *C. parvum* genome depth and breadth for diluted DNA samples prepared with iTru/NEB libraries normalized by 100,000 reads, (B) species assignment of diluted *C. parvum* samples prepared with iTru/NEB protocol and unenriched (*U*) or single-enriched (*S*) mapped to the ten *Cryptosporidium* genome sequences reference (10-CrypGS); (C) *C. parvum* genome depth and breadth for diluted DNA samples prepared with iNext/NextEra libraries normalized by 100,000 reads, (D) species assignment of diluted *C. parvum* samples prepared with iNextEra protocol and unenriched (*U*), single-enriched (*S*), or double-enriched (*D*) mapped to the ten *Cryptosporidium* genome sequences reference (10-CrypGS). In the species assignment plots, the first letter in the sample name corresponds to the library type, and the second letter corresponds to the level of input of DNA (A-G). The direction of the arrow indicates high to low input (A → G).

We tested different levels of input DNA with serial dilutions to determine the limit of detection (LOD) of target genomic DNA using both iTru and iNextEra libraries. For iTru libraries (Supplementary Notes #10.1), the percentage of reads mapped per sample to *C. parvum* positively correlated with the initial concentration of DNA. That is, with low inputs of 0.0001 ng, 0.00001 ng, and the negative control (0 ng), low percentages of reads mapping were obtained. Statistically significant differences in the percentage of reads mapped were found between 1 ng and values of 0.01 ng and below (p-values ≦ 0.004) and 0.1 ng and 0.01 ng and below (p-values ≦ 0.040; Fig. S17). The depth of *C. parvum* genome coverage per 100,000 reads was below an average of ~1X for dilutions equal to or below 0.01 ng, 2.2X for 0.1 ng, and 2.5X for 1 ng, with significant differences between the means of 1 ng and 0.1 ng and all other dilutions (p-values < 0.0001; Fig. 4A; not normalized values Fig. S18). The breadth of genome coverage normalized per number of reads did not correlate with input (Fig. 4A; Supplementary Results), with significant differences between the input of 0.01 ng, which had a high percentage of breadth (14.7%), and all other comparisons (Supplementary Results; not normalized values Fig. S18). When mapped to the 10-CrypGS, we observed that for unenriched samples, accurate detection is observed only for 1 ng and 0.1 ng input. Lower concentrations show the effects of background DNA (Fig. 4B, unenriched). For single enrichment libraries, we observe that *C. parvum* is detected at high proportions in inputs of 0.001 ng or higher (Fig. 4B, single enrichment, samples A-D), with low inputs (samples E-G) showing background effects.

For the dilution experiment with iNextEra libraries, we observed that the number of raw reads obtained varied among library types and dilutions (Supplementary Notes #10.2; Table S15, Fig. S19). Regardless of library type, we found that the average percentage of reads mapped to the *C. parvum* genome increased with input, ranging from 0.03% in the negative control to 55.39% with 1 ng of input (Fig. S20). The percentage of mapped reads was lower for inputs of ≤ 0.01 ng vs. 1 ng (p-values ≤ 0.015) and for inputs ≤ 0.001 ng vs. 0.1 ng (p-values ≤ 0.05). The depth and breadth of *C. parvum* genome coverage increased as input DNA increased (Fig. S21). However, only the breadth of genome coverage between the two highest inputs (1 ng and 0.1 ng) differed from all other input samples (p-values ≤ 0.017). When normalizing by the number of retained reads, depth and breadth are significantly higher when 1 ng was compared to < 0.01 ng inputs (p-values ≤ 0.034, Fig. 4C), and the comparisons of 0.1 ng with 0.001 and negative control (p-values ≤ 0.046). When reads were mapped to 10-CrypGS, the correct assignment of reads was highly correlated with input DNA (Fig. 4D). Accurate detection is observed in unenriched samples with inputs ≥ 0.1 ng, in single-enriched samples with ≥ 0.01 ng, and in double-enriched samples consistently with ≥ 0.001 ng, and less consistently in samples of 0.0001 ng (Fig. 4D).

### LOD: In Vitro Sensitivity Test with Different Dilution Levels of Baits

We tested a series of bait dilutions (1/2 – 1/16) to determine the minimum quantity of baits that could be used in a capture reaction to achieve a similar performance as the undiluted baits, using both iTru and iNextEra libraries. For iTru libraries, no difference was observed in bait dilution for the number of reads obtained (Supplementary Notes #11.1; p-values ≥ 0.975; Fig. S22; Table S14) or the percentage of reads mapping to *C. parvum* (Fig. S23). The depth and breadth of genome coverage decreased slightly as the bait dilution progressed; however, these differences were not statistically significant (p-values ≥ 0.586; Fig. S24).

Based on the results above, the experiments with iNextEra libraries focused on baits with 1/4 and 1/8^th^ dilutions (Supplementary Notes #11.2; Table S15). The number of raw reads obtained was similar for both bait dilutions and unenriched (Fig. S25). The percentage of mapped reads was correlated with the bait input amount, averaging ~18% for the enriched libraries and 0.11% for unenriched libraries (Fig. S26, p-value ≥ 0.337). The depth and breadth of *C. parvum* genome coverage were, on average, 43.9X and 41.67% for libraries enriched with quarter dilutions of the baits, 11.8X, and 20.64% for libraries enriched with an eighth dilution of the baits, vs. 0.03X and 1.55% for unenriched libraries, however, values were not significantly different, even after normalization based on retained reads (all p-values ≥ 0.091; Figs S27–28).

### Enriching Seven Human Fecal DNA samples with the CryptoCap_100k Bait Set

For libraries from DNA obtained from seven clinical patients’ fecal samples, roughly a similar average number of reads were obtained for unenriched and enriched libraries (Supplementary Notes #12.1, Table S16, Fig. S29). The percentage of reads mapping to 10-CrypGS increased from 0.02–0.15% (unenriched) to 0.37–18.17% (enriched), with a fold change of 9.25–84.5X for the four DOCK samples and 354–479X for the three UK samples (Table S16, Fig. S30, p-value = 0.02). Species assignment using 10-CrypGS shows drastic differences between enriched and unenriched libraries (Fig. S31); all clinical samples from the unenriched libraries show a high proportion of the few mapping reads assigned to non-human infecting *C. muris* (Fig. S31 right), similar to what is observed as background effect from samples with low concentration in the LOD experiment. In contrast, DOCK and UKH101 enriched libraries yield reads assigned mostly to *C. hominis* and *C. cuniculus*. Samples EC4 and UKP196 contain high proportions of *C. parvum* (Fig. S31 left). No reads mapped to the *gp60* database, and very few mapped to the *18S rRNA* database with assignment to species not typically observed in human infections (Fig. S32).

### Enriching 100 Human Fecal DNA Samples with the CryptoCap_100k Bait Set

#### iTru libraries

**Figure 5.**
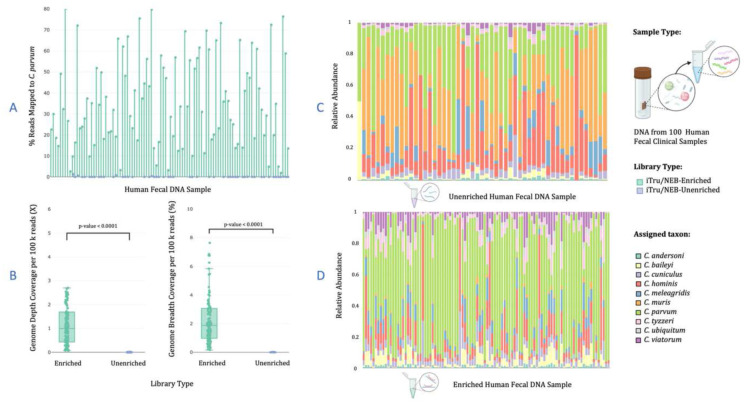
Summary statistics for sequence data from 100 clinical samples set prepared with the NEB protocol and iTru primers, comparing libraries enriched with CryptoCap_100k bait set versus unenriched libraries for validation. (A) Scatter plot of percentage of reads mapped from one round of enrichment compared to unenriched reads for each sample (x-axis labels not shown but in order from left to right sample 1 to sample 100), (B) Genome depth and breadth of mapped regions for each sample in enriched libraries compared to unenriched normalized by 100,000 reads, and (C) species assignment to each sample prepared with NEB protocol and enriched when mapped to a reference database containing ten *Cryptosporidium* genome sequences (10-CrypGS). (D) species assignment to each sample prepared with NEB protocol and left unenriched when mapped to a reference database containing ten *Cryptosporidium* genome sequences (10-CrypGS).

For libraries prepared from fecal DNA obtained from 100 patients with cryptosporidiosis using the iTru protocol, 26 samples required more than one attempt and an increased number of PCR cycles (from 14 to 18) at library preparation to produce a library that passed QC. We obtained an average of 2.6 million paired reads per enriched library for the 100 samples, and 4.1 million paired reads for 73 samples from unenriched libraries (Supplementary Notes #12.2.1, Table S17; Fig. S33). On average, 35.6% of enriched vs. 0.08% of unenriched retained reads mapped to the *C. parvum* genome, respectively (p-value < 0.0001; Fig. 5A; Table S17; Fig. S34). The libraries were enriched to *C. parvum* by an average factor of 2,095X (range 7.20 – 5,097X; Table S17). The depth and breadth of genome coverage were significantly higher for enriched samples with an average depth normalized per 100,000 reads of 1.12X and breadth of 2.25% for enriched vs. an average depth of 0.0004X and breadth of 0.0008% for the unenriched libraries (p-values < 0.0001; Fig. 5B; Table S17; not normalized values Fig. S35).

Next, we assessed species assignment from the relative number of hits from the iTru libraries to each species in 10-CrypGS. The percentage of reads mapping was positively but weakly correlated to the qPCR C_T_ values for unenriched libraries and negatively correlated with enriched libraries (Figs. S36–37, Supplementary Results). A 5.6% increase of paired reads mapping to 10-CrypGS vs. *C. parvum* genome was observed for enriched libraries and 0.05% for unenriched (Table S17). Still, only 53 unenriched samples mapped to the reference, with an average of 2,705 mapped reads per sample. The species assignments for unenriched libraries generally resemble the complex background used for the dilution experiments, including mostly hits to *C. muris* (cf. Fig. 4B and 5C). Following a single round of enrichment, an average of 1.03 million reads hit the database (Table S17), with most hits matching *C. parvum* (63 samples have >50% of hits to *C. parvum*; Fig. 5D), but many samples have moderate proportions of reads assigned to other species, including species that have limited evidence that they infect humans (Fig. 5D; see Supplementary Results). Thus, species assignments differed drastically between enriched vs. unenriched samples (Fig. 5C vs. 5D), with a significant shift in species assignments toward species with more evidence of human infection, except that a slightly lower proportion of reads are assigned to *C. hominis* (see Supplementary Results).

#### iNextEra Libraries

**Figure 6.**
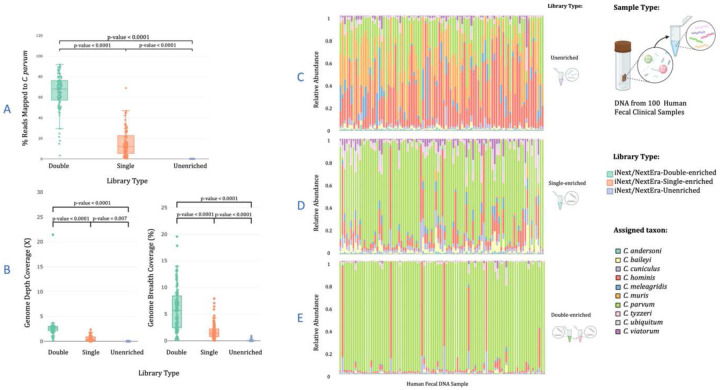
Summary statistics for sequence data from 100 clinical samples set prepared with the iNextEra protocol and iNext primers, comparing libraries enriched with CryptoCap_100k bait set versus unenriched libraries for validation. (A) Boxplot of percentage of reads mapped to *C. parvum* from double-enriched, singleenriched, and unenriched libraries, (B) Genome depth and breadth of mapped regions for each sample in double-enriched, single-enriched, and unenriched libraries normalized by 100,000 reads, (C) species assignment to each sample prepared with iNextEra protocol with nor further enrichment when mapped to a reference database containing ten *Cryptosporidium* genome sequences (10-CrypGS), (D) species assignment to each sample prepared with iNextEra protocol and single-enriched with CryptoCap_100K when mapped to a reference database containing ten *Cryptosporidium* genome sequences, (E) species assignment to each sample prepared with iNextEra protocol and double-enriched with CryptoCap_100K when mapped to a reference database containing ten *Cryptosporidium* genome sequences (10-CrypGS).

For the 91 clinical samples for which iNextEra protocol was used, we obtained an average of 3.7 million paired reads per unenriched library, 5.3 million paired reads for single-enriched libraries, and 3.6 million for double-enriched libraries (Supplementary Notes #12.2.2, Table S18; Fig. S38). On average of 0.03% of unenriched reads, 15.85% of single-enriched reads, and 64.98% of double-enriched reads mapped to the *C. parvum* genome (Supplementary Notes #12.2.2, all p-values < 0.0001, Fig. 6A, Table S18, Fig S39), yielding an average enrichment factor of 1,112X from unenriched to single-enriched, and an extra 9.24X from single- to double-enriched (Table S18). The depth and breadth of genome coverage normalized per 100,000 reads were significantly higher for double-enriched samples, followed by single-enriched samples (Fig. 6B). The depth of genome coverage per 100,000 reads was for unenriched = 0.001X, single-enriched = 0.563X, and for double-enriched = 2.70X. The breadth of genome coverage was for unenriched = 0.060%, for single-enriched = 1.79%, and for double-enriched libraries = 5.99% (Fig. 6B, Table S18, not normalized values Fig. S40). The percentage of reads mapping to the 10-CrypGS was negatively correlated to the qPCR C_T_ values for all library types. Double-enrichments allow retrieving a higher percentage of target reads at higher Ct values than the other two library types (Figs. S41-S43, Supplementary Results). Species assignments were similar to those from iTru libraries in unenriched and single-enriched libraries (cf. Figs. 5C vs. 6C, and Figs. 5D vs. Fig. 6D). The species assignment analysis shows a substantially increased proportion of hits to the most frequently observed human-infecting species, *C. parvum*, and *C. hominis* in double-enriched libraries compared to single- and unenriched libraries (Fig. 6E).

#### iNextEra vs. iTru Libraries

iNextEra and iTru libraries performed similarly (Table S19) but with noteworthy differences. For single-enriched libraries, we obtained an average of 2.04X more read pairs for iNextEra than iTru libraries (Supplementary Notes #12.2.3, Table S19; Fig. S44). More reads were retained following quality filtering for iNextera vs. iTru, but this result is correlated with sequencing instruments used (iNextEra used NovaSeq X vs. iTru used HiSeq X). A higher percentage of reads mapped to the *C. parvum* reference in samples from iTru vs. iNextEra libraries (35.6% vs. 15.9%, respectively; p-value < 0.0001; Fig. S45), which resulted in iTru libraries yielding an average enrichment factor about twice as large as iNextEra libraries (Table S19). However, the normalized depth of coverage is very similar for both protocols (0.5X, p-value = 0.7718; not normalized values Fig. S46). The normalized breadth of coverage was significantly higher for the iNextEra libraries (p-value < 0.0001; not normalized values Fig. S47, both normalized Fig. S48).

## Discussion

Due to the limitations of currently available methods employed to study *Cryptosporidium* spp., there is an overall lack of understanding of *Cryptosporidium* biological and genomic diversity and how it correlates with transmission, infection, pathogenesis, and host response. Here, we developed and critically evaluated a capture system to obtain *Cryptosporidium* DNA from samples with low input DNA levels, varying target concentrations, and varying species composition to generate genomic data from human-infecting *Cryptosporidium* species. Our bait capture assay increases on-target reads by 2–3 orders of magnitude, enables the pooling of ~10 libraries per capture reaction, and the dilution of the baits, thus significantly increasing the affordability of use in future studies (see details below). Our study revealed several significant findings: (i) CryptoCap_100k is highly specific and achieves a higher percentage of on-target reads than unenriched libraries in clinical samples with both iTru (~437X increase) and iNext (~500X in single-enriched and ~2,167X in double-enriched) library preparation methods, (ii) in DNA samples from pure oocysts or manufacturer, CryptoCap_100k can cover 99% of the genome without deep sequencing, and (iii) in real-world human fecal DNA samples, CryptoCap_100k recovers sequences for an average of about half of the genome, but with a relatively wide range, including samples with high Ct values, and (iv) double enrichments increase the number of on-target reads even further and provide substantially better species assignment.

This study was not the first to apply bait capture to whole genome sequencing of human-infecting *Cryptosporidium* spp^39^. However, our study is the first to design baits based on new and more complete genome sequences (e.g.*, C. parvum* IOWA-ATCC^42^) and to include baits to account for variation among species of human-infecting *Cryptosporidium* spp. by selecting for unique regions of these genomes when compared to the *C. parvum* genome sequence. Thus, our new bait set could yield sequences from variable regions within and among species of *Cryptosporidium* while being highly specific, as shown by the low levels of cross-mapping to other non-*Cryptosporidum* species. In addition, our study innovates using *in silico* testing to evaluate baits on multiple species of *Cryptosporidium* and provide specific details on the expected performance of the hybridization bait capture assay. In clinical samples, our study finds that CryptoCap_100k provides a higher percentage of target reads (Fig.5A and 6A) and higher depth and breadth of genome coverage (Fig. 5B and 6B) across all samples compared to unenriched libraries. These findings are comparable to our *in silico* results (Table S1, Fig. 1). Thus, the CryptoCap_100k bait set can cover 99% or more of the *Cryptosporidium* spp. genome *in silico* and ~ 50% *in vitro* in samples single-enriched with iNextEra with Ct values ranging from 18.01 to 31.08 (Table S19).

Studies on the population genetic structure of *Cryptosporidium* spp. are needed, but obtaining pure and sufficient *Cryptosporidium* DNA for validation and testing is challenging^43^. Purifying oocysts can be labor-intensive and time-consuming, require specialized equipment, and may result in significant loss of oocysts and DNA^44^. As one of our goals, we aimed to remove the need to purify oocysts to obtain usable genomic data. To make recommendations about how to best use our CryptoCap_100k baits, we define two distinct limits of detection (LOD): (i) the LOD for minimum target genomic equivalents and (ii) the LOD for the minimum bait concentration used in the assay. We diluted our pure *C. parvum* DNA in a complex background community to reflect accurate environmental or clinical samples and determine the threshold at which we no longer obtain quality data for our enrichment libraries. Departing from the fact that a single haploid sporozoite contains a little over ~9 fg of DNA. Our LOD for *C. parvum* assay using both the NEB and iNextEra enriched libraries found that we obtained a high percentage of target reads, substantial breadth and depth of genome coverage, and accurate species assignment with inputs as low as 0.01 ng (~1,000 sporozoites or ~250 oocysts equivalents) of *C. parvum* DNA (Figs. 4A–4B). Moreover, we were able to improve all these parameters further in double-enriched iNextEra libraries that contained 0.001 ng (~100 sporozoite or ~25 oocyst equivalents) of *C. parvum* DNA (Fig. 4D). In comparison, the unenriched libraries for both the NEB and iNextEra preparation methods had extremely poor species assignment when input was below 0.1 ng (Fig. 4B & Fig. 4D). These findings establish that our CryptoCap_100k baits can obtain ample genomic information at low levels, thus eliminating the need for purifying oocysts to gain useful information. When assessing the LOD for the CryptoCap_100k baits, we found that there was no significant difference in the percentage of reads that mapped, depth and breadth of genome coverage observed between the different bait dilutions (full down to a sixteenth, Figs. S24-S25), still we observed a trend where a higher percentage of target reads and breadth were obtained at quarter dilutions and higher (Figs. S26-S27). Our findings demonstrate that the baits can be diluted to save on cost.

Clinical human stool samples provided for testing are characterized by a small volume containing a low overall abundance of *Cryptosporidium* oocysts in a large background community of non-target organisms. This problem is heightened by the lack of a readily available *in vitro* culture system^43,45,46^. We successfully tested our CryptoCap_100k baits on an initial small set of seven samples. Once the approach worked well (Fig. S31), we proceeded with the larger set of 100 clinical samples. We observed significantly more reads mapping to *Cryptosporidium* genome sequences in enriched vs. unenriched libraries with both the NEB and iNextEra library methods (Fig. 5A; Fig. 6A). Furthermore, the average breadth and depth of genome coverage for the 100 clinical samples were significantly higher for both enriched library preparation methods compared to unenriched (Fig. 5B; Fig. 6B). This demonstrates an increased level of on-target reads for both library preparation methods, leading to better species assignment of the reads (Figs. 5C–D, Figs. 6C–E). Several studies recommend only sequencing samples with Ct values < 16^44,47^. However, even with an average large Ct value of 25.4, we still see, on average, 31.3% breadth and 28.1X depth in the clinical samples using NEB libraries and 54.38% coverage and 32.11X depth in the clinical samples using iNextEra libraries (Table S17-S18). Our linear regression model estimated that the percentage of reads mapped against 10-CrypGS decreased by only 2.8% for each increment of Ct units for NEB-enriched libraries (Fig S36). For iNextEra libraries, unenriched libraries show 0% target reads at a Ct value of 35 (Fig. S41), and single-enriched libraries show 0% target reads approx. at 40 Ct (Fig. S42), and double-enriched libraries display a flatter trend, indicating that even high Ct-value samples tend to retain high percentages (Fig. S43). Following this trend, double enrichments with iNextEra library preparation always resulted in significant percentages of reads mapped to the *C. parvum* genome and the depth of the genome covered (Fig. 6A–B), as well as more accurate species assignment from the reads obtained (Figs. 4D & 6E). Species assignment following enrichment coincides with the species detected in these same samples using *C. hominis* and C*. parvum* species-specific duplex real-time PCR assay^48^. Double enrichment could be especially useful when seeking accurate information from samples believed to be a new strain or species or to increase the confidence in variations found in the genome by increasing the depth of sequencing coverage. Additionally, the increase in on-target reads and depth could aid researchers in parsing mixed infections.

Clinical *Cryptosporidium* infections can contain multiple strains, subtypes, or species within one infection. Current methods for detecting mixed infections include amplicon, PCR-RFLP, and MLST approaches^21,49,50^. However, these methods have inherent biases due to preferential amplification and can be difficult to execute for mixed infections that are not comprised of *C. parvum* and *C. hominis*^21,22,49^. We simulated *in vitro* a mixed infection using two DNA samples of *C. parvum* and *C. meleagridis* pooled at planned equal proportions. We found a slightly higher proportion of *C. parvum* than *C. meleagridis* in unenriched and enriched reads. These differences may be due to pipetting errors or discrepancies in the abundance of the target in the two DNA samples, thus creating unequal competition. However, we demonstrated that in the case of a mixed infection, two species of *Cryptosporidium* were captured and identified, though the subtle increase in the proportion of *C. parvum* in the enriched pool could also suggest that enrichment could be slightly biased (Fig. 3A). This could be expected as *C. parvum* was the base species used for bait design, however, such biases were not conspicuous in the mixed-infection computer simulations (Fig. 2) nor in clinical samples that showed *C. hominis* assignments. Potential mixed infections were detected in EC4 (Fig. S31) and UKH151 (Fig. 6E). Overall, our CryptoCap_100k baits can readily detect mixed species infections and can obtain estimates without extreme skewing of the relative abundances, except perhaps for *C. hominis* and *C. cuniculus*, in which their introgression history makes it challenging when using our current 10-CrypGS database. Further updates of databases for species assignment are necessary tools for the community to help overcome this issue. Our bait set provides the ability to generate data that could be used not only for insights into the genome sequences of lesser sequenced species or subtypes but also to populate these databases and refine analyses.

Our assay can be implemented in various scenarios, and the cost savings will vary depending on the abundance of *Cryptosporidium* DNA in the sample. To illustrate costs, please see Supplementary Results and Table S20. In brief, our assay reduces the cost of characterizing genome-wide *Cryptosporidium* DNA sequences in fecal samples by an average of about 98% (Table S20, Supplementary Results).

In conclusion, our findings demonstrate the ability of the CryptoCap_100k bait set to cover >80% of the genome with high depths *in vitro* in pure samples (Figs. S13-S14) and achieve as high as 3,690X fold-change enrichment in clinical samples (Table S18), and as a result high accuracy of species assignment. Studies using the CryptoCap_100k bait set can improve our understanding of the population genetics of human-infecting *Cryptosporidium* species and their association with epidemiology. Finally, generating economical and efficient genome-level data allows us to grow our databases and refine analyses and methods.

## Methods

### CryptoCap_100k Bait Design & Characterization

Based on sequence similarity and availability on CryptoDB.org, six species’ genomes of *Cryptosporidium* were chosen for bait design (*C. parvum*, *C. hominis*, *C. cuniculus*, *C. tyzzeri*, *C. meleagridis*, and *C. viatorum*), along with 55 *18S rRNA* sequences and eight *gp60* sequences (see Data Availability). All sequences were soft-masked for simple and low-complexity repeats using RepeatMasker v. 4.1.1 (http://www.repeatmasker.org/). From all sequences, we aimed to design 100,000 120-nucleotide baits. Only baits that were ≦50% soft-masked for repeats were retained. Applying the design configuration to all six genome sequences resulted in 435,323 raw baits. Then, VSEARCH v. 2.15.0^51^ was used to cluster baits at various levels of sequence overlap and identity (Table 1). This approach used tolerance for mismatches between the bait and target sequence to reduce the number of baits while maintaining diversity. Furthermore, VSEARCH uses a “greedy algorithm,” which results in bias based on the order of the input sequences. This was used to the advantage of the design by selecting the genome order for each bait set to be clustered to give preference to specific genome sequences based on completeness and importance. The input order was *C. parvum*, *C. hominis*, *C. cuniculus*, *C. tyzzeri*, *C. meleagridis*, and *C. viatorum*. *C. parvum* IOWAATCC was used as the alignment reference (listed first) for all clustering procedures: *C. hominis*, *C. cuniculus*, and *C. tyzzeri* were collapsed at 50% overlap, and 90% identity relative to *C. parvum*; *C. meleagridis* and *C. viatorum* were collapsed at 45% overlap and 85% identity relative to *C. parvum*. Due to the high number of baits obtained from *C. viatorum*, a subset was randomly removed. Raw 403 baits were obtained for the *18S rRNA* targets; we collapsed these at 50% overlap and 95% identity to arrive at the final set.

After bait design, we characterized the CryptoCap_100k bait set by using twelve genome sequences available for ten species of *Cryptosporidium* (please see Data Availability). BWA v 0.7.17^52^ was used to map the CryptoCap_100k bait set to each genome sequence. Alignment files were generated and converted using Samtools v.1.10^52,53^. Then, the bamCoverage function from DeepTools v. 3.3.1^54^ was used to produce a bigwig file, which generated a coverage track calculated as the number of reads per bin, where bins are short consecutive counting windows of a defined size of 50. Then, bigwig files were visualized in IGV-Web app version 1.10.8^55^. To obtain alignment statistics, the depth option from Samtools v1.1.0 and the -*a* flag were used to get all sites and create a depth file^53^. A custom awk script (Supplementary Notes #1) obtained the mean depth and proportion of baits mapped to each reference. The breadth of coverage on each genome was obtained by estimating the number of bases covered in the alignment over the reference length when setting a minimum coverage depth of 4.

We also compared the CryptoCap_100k bait set to CryptoCap_73K^39^, which according to our analyses (Supplementary Notes #2), contains 68,966 baits of 120 nt. We estimated the same parameters above using the identical genome sequences for this bait set, and we made further statistical comparisons using Welch’s two-sample t-test.

### In Silico Simulations of Enrichment with CryptoCap_100k & Sequencing

In a simple infection scenario, we used the twelve available genome sequences for ten *Cryptosporidium* species to simulate the enrichment and sequencing process with the CryptoCap_100k bait set (Supplementary Notes #3). Each genome was used as if it belonged to a sample containing only such a genome. First, we obtained the coordinates of the above-mapped baits on each of the twelve genome sequences (*CryptoCap_100k Bait Design & Characterization*). To simulate the hybridization process, we extended those mapping positions to several bases to the left and right, assuming that the bait was annealing to the centroid of larger fragments being captured. We performed two types of extensions to understand the effect of mean library fragment size on enrichment and data. We thus simulated the capture of fragments with two different size distributions. We took the first mapping positions for all baits and extended each region for large fragments, 200 bases to the left and 320 bases (120 bait +200) to the right, to simulate a library insert size of 520 bp. Also, we extended the coordinate where the bait mapped 40 bases to the left and 160 bases (120 bait + 40) to the right to simulate a library insert size of 200 bp. Then, the faidx option from Samtools was used to extract the two types of extended sequences from each genome to create a fasta file^53^.

To simulate the sequencing process from these “captured” fragments, the software ART 2016.06.05^56^ was used to reproduce ~200,000 paired-end Illumina 150 bp fastq reads from each fasta. Each set of fastq files was mapped to the respective genome sequence using BWA v 0.7.17^52^, and we estimated depth and breadth coverage from these reads on each genome and produced bigwig files for visualization of the results.

Then, we assessed and validated the simulated data species assignment accuracy (Supplementary Notes #4). To do this, we used three reference databases. First, we generated one fasta file containing ten *Cryptosporidium* genome sequences that served as a reference (10-CrypGS, from the twelve genome sequences reference, we removed *C. hominis* UdeA01 and *C. parvum* IOWA II). Second, we used a database of *gp60* sequences, the most widely used genetic marker for subtyping *Cryptosporidium*, which contains the different subtypes identified in human cases^15^. Third, we used a curated CryptoDB.org database of *18S rRNA*, the second most characterized loci in the group^57–59^. We used BBMap 38.90^60^ to map fastq reads to each of the three reference files, which by default allows multi-mapping (secondary alignment) of baits to the genomes. We only considered regions where both reads were mapped to count the number of hits. We plotted the proportion of paired hits to specific *Cryptosporidium* species for each simulated dataset.

To understand the specificity of the bait set, by the sensitivity of the baits to hybridize to off-target DNA, common in environmental and clinical samples, we assessed *in silico* the affinity of our baits to various genome sequences, including microbial, human, and cow sequences (Supplementary Notes #5 and #6). To do this, we used a fasta file with 1,008 whole genome sequences^41^, which includes genomes of bacteria, archaea, and eukaryote organisms, including the human genome. Separately, we also used the *Bos taurus* genome sequence as a reference (GCA_002263795.3) to assess if our baits can hybridize to DNA used as background for dilution *in vitro* experiments. For both analyses, metagenomes, and *Bos taurus*, we used BWA 0.7.17^52^ to map the baits. Then, we estimated the number of hits as well as the quality of the match.

Finally, to explore *in silico*, the ability to discriminate different species within a mixed-infection sample enriched with our bait set, we created four input files to simulate different mixed infections scenarios (Supplementary Notes #7). We did this by concatenating the fastq files already generated for each species above and using only those designed large insert fragments. The four input types were: i) a fastq file containing reads from all twelve genomes (*C. andersoni, C. baileyi, C. cuniculus, C. hominis* x 2*, C. meleagridis, C. muris, C. parvum* x 2*, C. tyzzeri, C. ubiquitum*, and *C. viatorum*); ii) a file containing the sequences of the ten representative species (*C. andersoni, C. baileyi, C. cuniculus, C. hominis, C. meleagridis, C. muris, C. parvum, C. tyzzeri, C. ubiquitum*, and *C. viatorum*); iii) a file containing the cluster of five species that share high percent identity (*C. parvum, C. hominis, C. tyzzeri, C. meleagridis*, and *C. ubiquitum*); and 4) a file containing the three most common species found in human infections (*C. hominis, C. cuniculus*, and *C. parvum*). Then, we mapped these mixed reads to 10-CrypGS, the *gp60* database, and the *18S rRNA* database following the same parameters as above.

### In Vitro Enrichment & Validation with CryptoCap_100k Baits with Pure Oocysts’ DNA

Pure oocyst DNA was obtained for three *C. parvum* samples from Dr. Michael Grigg (National Institute of Health, US) and a duplicate of *C. meleagridis* DNA isolate TU1867 from BEI Resources (BEI Resources NR2521 Manassa, VA). Metagenomic libraries were prepared with the New England BioLabs Ultra FS II library kit (please read *Metagenomic Libraries for In Vitro Experiments*), and an aliquot of these were enriched with CryptoCap_100k bait sets (please read *Hybridization Capture Enrichments*). Enriched and unenriched libraries were sequenced in an Illumina MiSeq using a PE250 kit.

### In Vitro Simulation of Mixed Infections

To test the ability to detect mixed infection samples, after NEB library preparation of DNA samples (please read *In Vitro Enrichment & Validation with CryptoCap_100k Baits with Pure Oocysts’ DNA*) and before hybridization bait capture, two library products were pooled to simulate a mixed infection. To accomplish this, one indexed library product from a pure sample from *C. parvum* and one with different indexes from *C. meleagridis* were pooled at equimolar ratios. After pooling, we divided the products into two aliquots, enriched one with CryptoCap_100k baits, and left the second unenriched. Then, cleaning and sequencing proceeded, as explained below. We predicted that the number of reads obtained based on the demultiplexing based on primers attached before enrichment should be roughly 50% for each species for each sample. These pooled libraries were sequenced in an Illumina MiSeq using a PE250 kit.

### In Vitro Test of Limits of Detection with Different Levels of C. parvum Dilution

To determine the sensitivity or limits of detection (LOD) of *Cryptosporidium* when using CryptoCap_100k bait set to mimic samples with a low concentration of target DNA, we performed two library preps, one experiment with NEB libraries and iTru primers^61^ and one experiment with iNextEra libraries and iNext primers^61^.

For the experiment with NEB libraries, we diluted pure *C. parvum* genomic DNA in a complex background community, such as a community of DNA molecules from calf thymus (Sigma-Aldrich, St. Louis, MO). For this experiment, a 45-ng aliquot of calf thymus DNA was pipetted into five tubes. In the first tube, 5 ng of *C. parvum* genomic DNA was added. Following this, three 1:10 serial dilutions were performed using the first tube as a starter. The fifth tube corresponded to a negative control, containing just the calf thymus. Then, metagenomic libraries with the NEB protocol and enrichments were prepared for these libraries (please read *Metagenomic Libraries for In Vitro Experiments* and *Hybridization Capture Enrichments*). Number of reads obtained, percentage of reads mapped to the corresponding genome, and depth and breadth of genome coverage were estimated.

For the experiment with iNextEra libraries, we diluted genomic DNA from pure preparations of *C. parvum* into the complex background community. For this experiment, 45 ng aliquots of calf thymus DNA were pipetted into six tubes. In the first tube, 10 ng of *C. parvum* genomic DNA was added. Four additional 1:10 serial dilutions were performed using the first tube as a starter. The sixth tube corresponded to the negative control, containing just the calf thymus. We grouped the six tubes in five different treatments: one unenriched, one single-enriched with one-quarter of the recommended baits, one single-enriched with one-eight of the recommended baits, one double-enriched with one-quarter of the recommended baits, and one double-enriched with one-eight of the recommended baits. Enrichments were prepared according to *Metagenomic Libraries for In Vitro Experiments* and *Hybridization Capture Enrichments*. The number of reads obtained, percentage of reads mapped to the corresponding genome, depth and breadth of genome coverage, and species assignment based on the mapping of reads against 10-CrypGS were performed to allow comparisons between dilutions.

### In Vitro Test of Limits of Detection with Different Levels of Bait Dilution

To determine the sensitivity or limits of detection (LOD) of *Cryptosporidium* when enriching libraries with the CryptoCap_100k bait set, we modulated the quantity of baits added to the capture reaction, as above, in one experiment with NEB libraries and one experiment with iNextEra libraries.

For both NEB libraries and iNextEra libraries, the bait dilution sensitivity assay performed considered pools of the dual-indexed metagenomic libraries from the dilutions experiment for *Cryptosporidium* plus the negative control (please see In Vitro Test of Limits of Detection with Different Levels of C. parvum Dilution). For NEB libraries, we tested five different bait quantities: full volume (5.5 μL, per the manufacturer’s specifications), half, quarter, eighth, and sixteenth. For iNextEra libraries, we tested quarter and eighth bait dilutions. In these assays, the enrichment procedure was followed according to the description in *Hybridization Capture Enrichments*, where the partial bait quantities were achieved by serially diluting baits with water to a half, quarter, eighth, and sixteenth of the concentration of the stock baits. The number of reads obtained, the percentage of reads mapped to the corresponding genome, and the depth and breadth of genome coverage were compared.

### In Vitro Enrichment & Validation with CryptoCap_100k Baits with Clinical Samples

Two clinical datasets were used for real-world validation steps. First, Dr. Michael Grigg (National Institute of Health, US) and Prof. Rachel Chalmers’s laboratory (Public Health Wales Microbiology and Health Protection, United Kingdom) provided DNA extracted from seven de-identified patient fecal samples. These seven samples were obtained on a NIAID IRB-approved protocol NCT00006150. The seven clinical samples were extracted using the Qiagen PowerSoil Kit (Qiagen, Hilden, German). For this set of samples, NEB library protocol was used, and aliquots were enriched with CryptoCap_100k bait sets (please read *Hybridization Capture Enrichments*). Enriched and unenriched libraries were sequenced in an Illumina HiSeq using a PE150 kit (300 cycles).

Second, 100 DNA extractions (Qiagen QIAamp Fast DNA Stool kit) from clinical samples from Prof. Rachel Chalmers’s laboratory (Public Health Wales—PHW— Microbiology and Health Protection, United Kingdom) were used. This work was carried out in accordance with a material transfer agreement between UGA and PHW for the analysis of de-identified *Cryptosporidium* DNA, which did not require ethical approval. A real-time qPCR assay was used to quantify the detection of *Cryptosporidium* species from these clinical samples, using primers for the *18S rRNA* gene and probes slightly modified from^62^. Here, we used as forward primer 5′ -GGGTTGTATTTATTAGATAAAGAACCA- 3′, reverse 5¢AGGCCAATACCCTACCGTCT-3′, and an internal probe 5′-TGACATATCATTCAAGTTTCTGAC-3′. Amplifications were carried on a final volume of 20 μL, which included 5 μL of 4x TaqPath^™^ 1-step RT-qPCR Master Mix (Thermo Fisher Scientific, MD, USA), 1 μL of 10 μM forward primer, 1 μL of 10 μM reverse primer, 2 μL of 2.5 μM fluorescent probe, 5 μL of 1:5 diluted DNA, and 6 μL of molecular grade water. Two genomic library protocols were prepared for each (NEB and iNextEra; please read *Metagenomic Libraries for In Vitro Experiments*). For both sets and types of libraries, aliquots of these libraries were enriched with CryptoCap_100k bait set; specifically, for iNextEra libraries, we also performed additional double enrichments (please read *Hybridization Capture Enrichments*). Enriched and unenriched libraries were sequenced in an Illumina NovaSeq using a PE150 kit.

### Metagenomic Libraries for In Vitro Experiments

Metagenomic libraries were prepared using two methods. First, New England BioLabs (NEB) Ultra FS II library kit (New England BioLabs, Ipswich, MA) was used. Enzymatic fragmentation of DNA extracts was performed according to the manufacturer, and it lasted between 5 to 11 minutes, based on the integrity of DNA visualized in a gel. Ligation products were cleaned in 1X SpeedBeads^63^. A 12- or 14-cycle PCR was used to add unique dual-indexes to each sample with iTru primers^61^. Samples were quantified with a Qubit 2.0 Fluorometer DNA high-sensitivity assay kit (Thermofisher, Waltham, MA) and cleaned with 1.25x SpeedBeads.

We implemented iNextEra library preparation protocols to decrease the time, cost, and amount of input DNA needed to produce libraries that would pass QC. Nine samples were depleted when producing iTru libraries and had no DNA remaining for iNextEra libraries. The switch from iTru to iNextEra also coincided with a switch in Illumina sequencing instruments, with the iNextEra libraries being run on the newly released Illumina NovaSeq X. For those samples prepared following iNextEra Library Prep, in brief, sample DNA was normalized to 10 ng/μL, for which 2.5 μL (25 ng) was used for a tagmentation reaction that included 3 μL of 2X TMP buffer and 0.5 μL of Transposome (BLT; CAT # 20015880, Illumina Inc.), incubated in a thermocycler at 53 °C for 30 minutes with the lid set at 80 °C. For PCR of the tagmentation products, a reaction containing 9.5 μL of Q5 Reaction Buffer, 1.25 μL of dNTPs 10 mM, 0.5 μL of Q5 High Fidelity Polymerase (New England Biolabs M0491L), 40.25 μL of nuclease-free water, 2.5 μL of iNext5 indexed primer at 5 μM, and 2.5 μL of iNext7 indexed primer at 5 μM^61^, and 6 μL of tagmented product. The cycling conditions for PCR were: 3 minutes at 72 °C, 3 minutes at 98 °C, followed by ten cycles of 98 °C for 45 seconds, 62 °C for 30 seconds, and 72 °C for 2 minutes, with a final extension at 72 °C for 1 minute. Products were validated in agarose gels and pooled with 5–7 other libraries at equimolar concentrations. Pools were then cleaned with 1–1.25X SpeedBeads (Sigma Aldrich, St. Louis, MO) and quantified with a Qubit 2.0 Fluorometer DNA (Thermofisher, Waltham, MA). Cleaned pools were then aliquoted to have an aliquot of an unenriched pool and an aliquot for enrichment with the CryptoCap_100k bait set.

### Hybridization Capture Enrichments with the CryptoCap_100k Bait Set

For different experiments, the input concentration per pool varied (i.e., for clinical samples using iTru/NEB, the input was between 25–80 ng/μL; for clinical samples using iNext/iNextEra, it was 103 ng/μL; and for the LOD experiment it was between 26–59 ng/μL). The enrichment process in each pool followed manufacturers’ protocols (mybaits v5, Daciel Arbor Biosciences, Ann Arbor, MI), using the standard protocol, with wash and hybridization temperatures of 63 °C and with modifications of bait quantities as described above. After enrichment, A P5/P7 PCR of 16–22 cycles was performed, followed by a 1–1.25X SpeedBeads clean-up (Sigma Aldrich, St. Louis, MO).

For the 100-clinical sample set and the LOD experiments, a second enrichment (double enrichment) of the iNextEra single-captured pools was performed. For these, aliquots of the cleaned single-captured pools were used independently as input for a second capture that followed all manufacturers’ steps from above, except that the volumes and the amount of baits were brought to one-quarter of the recommended. For post-double-enrichment P5/P7 PCR, 12–16 cycles were performed, followed by 1X SpeedBeads clean-up. All PCRs were performed using the KAPA HiFi Kit (Roche, Basel, Switzerland).

### In Vitro Bioinformatic Processing and Analyses

Bioinformatic and data analysis code, including custom scripts, are available in Supplementary Notes. All sequencing data were processed using Trimmomatic v. 0.39^64^ to remove Illumina TruSeq adaptors, low-quality bases and short reads. Trimmed retained reads were then mapped to specific genomes or databases using BBMap 38.9^60^. Samtools^53^ was used to sort bam files. Custom scripts were used to determine mapping statistics such as genome depth and breadth of coverage and count the number of hits against specific genomes.

Cleaned sequence reads from pure oocysts library samples and mixed-infection samples were mapped to the corresponding target genome (i.e., pure *C. parvum* samples to *C. parvum* IOWA-ATCC, pure *C. meleagridis* samples to chromosome-level assembly^65^, to 10-CrypGS, to the *gp60* database, and to the *18S rRNA* database (used in simulations). The sequencing reads from the LOD dilution experiments (i.e., input DNA and bait dilutions) were mapped to *C. parvum* and 10-CrypGS. The cleaned sequence reads from clinical samples were mapped to *C. parvum* IOWA-ATCC and to 10-CrypGS.

Data analyses were performed in R Studio 2023.06.0. Least-square comparisons of the means between experimental groups (e.g., unenriched and enriched, single-capture vs. double-capture, etc.) were performed to calculate the p-value using a confidence interval of 0.95 with the package: *lsmeans*^66^, which provides a t-test output when comparing two variables, or performs a Tukey method adjustment when comparing a family of three or more estimates. Other packages used were *tidyverse*, *ggplot*, and *plotly*^67^. Additionally, qPCR Ct values obtained were then used in the *parsnip 1.1.1* R package^68^ to define a regression model to predict the percentage of reads mapped against 10-CrypGS using a linear function.

## Figures and Tables

**Table 1.** Summary of *Cryptosporidium* species considered, genome version used, and criteria for designing CryptoCap_100k bait set.

Identifier	Reference Genome	Criteria (Identity: Overlap)	Number of Baits
**Whole Genome**			
*C. parvum*	GCA_015245375.1	Reference	73,672
*C. hominis*	GCA_001483515.1	90%:50%	2,851
*C. cuniculus*	GCA_004337835.1	90%:50%	738
*C. tyzzeri*	GCA_007210665.1	90%:50%	858
*C. meleagridis*	GCA_001593445.1	85%:45%	9,034
*C. viatorum*	GCA_004337795.1	85%:45%	11,774
***C. viatorum* (select genes)**		50%:95%	943
***18S rRNA***		50%:95%	63
***gP^60^***		NA	67
		**Total Number of Baits**	100,000
		GC Content	30.1%
		Soft Masked	2.8% (baits ranged from 0–50% masked)

**Table 2.** Bait characterization for CryptoCap_100k and CryptoCap_73k by mapping all baits to all available reference genome sequences. Mean depth, percent breadth of genome coverage, and percentage of baits mapped to each species’ genome sequence. We break down by species included and excluded from bait design.

		CryptoCap_100k 100,000 baits (This study)			CryptoCap_73k 68,966 baits (Khan and Alves-Ferreira et al., 2024)		
Species Information		Mean depth	% Breadth of coverage	% Baits mapped	Mean depth	% Breadth of coverage	% Baits mapped
Species included in CryptoCap_100k bait design	*C. parvum* IOWA II	1.086	97.55	84.98	0.909	90.89	100
	*C. parvum* IOWA ATCC	1.089	97.83	85.41	0.907	90.63	99.96
	*C. hominis* UdeA01	1.095	97.75	85.36	0.904	90.33	99.31
	*C. hominis* 30976	1.097	97.88	85.64	0.903	90.25	99.39
	*C. cuniculus* UKCU2	0.853	77.86	70.09	0.751	74.93	86.50
	*C. tyzzeri* UGA55	1.074	96.75	83.66	0.900	89.95	98.75
	*C. meleagridis* UKMEL1	0.997	91.42	80.45	0.793	79.23	91.27
	*C. viatorum* (partial inclusion) ABER_CVIA_1.0	0.714	66.68	60.99	0.529	51.40	64.36
Species not included in CryptoCap_100k bait design	*C. andersoni* 30847	0.008	0.52	1.08	0.005	0.45	0.87
	*C. baileyi* TAMU-09Q1	0.030	2.84	3.45	0.025	2.43	3.70
	*C. muris* RN66	0.008	0.60	1.12	0.005	0.47	0.91
	*C. ubiquitum* 39726	0.262	25.39	24.81	0.239	23.86	31.69

**Table 3.** Summary statistics of mapping *in silico* simulated enrichments with CryptoCap_100k baits of 150 bp fastq pairedreads for each of the twelve genomes assuming two input fragment size classes (200 bp and 520 bp). These mappings were only against each of the corresponding genomes.

Species Information		Fragment Size	# Simulated paired-reads	Mean read depth	Breadth of coverage
Species included in design	*C. parvum* IOWA II	~200 bp	255,486	8.42	99.1%
		~520 bp	255,332	8.42	99.6%
	*C. parvum* IOWA ATCC	~200 bp	256,947	8.45	99.4%
		~520 bp	256,947	8.45	99.9%
	*C. hominis* UdeA01	~200 bp	256,179	8.50	99.4%
		~520 bp	255,634	8.48	99.8%
	*C. hominis* 30976	~200 bp	257,208	8.52	99.6%
		~520 bp	257,102	8.51	99.9%
	*C. cuniculus* UKCU2	~200 bp	174,845	5.71	74.7%
		~520 bp	185,561	6.07	74.5%
	*C. tyzzeri* UGA55	~200 bp	249,737	8.31	98.4%
		~520 bp	248,498	8.27	98.9%
	*C. meleagridis*	~200 bp	241,764	8.07	98.5%
		~520 bp	241,770	8.08	99.9%
	*C. viatorum* ABER_CVIA_1.0	~200 bp	243,998	7.92	83.7%
		~520 bp	243,985	7.92	97.4%
Species not included in design	*C. andersoni* 30847	~200 bp	209,400	7.37	1.4%
		~520 bp	214,247	7.53	3.3%
	*C. baileyi* TAMU-09Q1	~200 bp	199,404	7.10	7.0%
		~520 bp	200,042	7.12	15.3%
	*C. muris* RN66	~200 bp	198,254	6.39	1.8%
		~520 bp	197,720	6.49	3.7%
	*C. ubiquitum* 39726	~200 bp	198,484	6.65	42.6%
		~520 bp	198,485	6.65	69.0%

**Table 4.** Summary statistics from enriching 100 human fecal DNA samples with two library preparation methods using CryptoCap_100k baits and obtaining Illumina PE150 reads. Source tables are Tables S17 and S18.

	iTru Libraries			iNextEra Libraries		
Average values	Not enriched	Single enriched		Not enriched	Single enriched	Double enriched
% retained after quality filtering	61.53	83.87		89.70	90.79	94.48
% reads on target *C. parvum*	0.04	35.62		0.033	15.85	64.98
% reads on target 10 genomes	0.11	41.23		0.100	17.90	65.78
Enrichment factor *	N/A	2,095		N/A	1,112	10,700**
Depth per 100k *	0.00	1.12		0.001	0.563	2.702
Breadth per 100k *	0.00	2.25		0.065	1.79	5.99
Number of samples	73	100		91	91	91

* (matching *C. parvum*)

** This value corresponds to the enrichment factor of double-enriched samples compared to not-enriched counterparts, which is not displayed in Tables S17 and S18.

## Data Availability

The six genome sequences that supported the CryptoCap_100k bait design were from GenBank: GCA_015245375.1, GCA_004337795.1, GCA_001483515.1, GCA_007210665.1, GCA_004337835.1, and GCA_001593445.1. The *18S rRNA* sequence data that supported the CryptoCap_100k bait design were from GenBank with the EU250845, LC089976, DQ288166, EF641022, GU951714, AF108864, LC012016, GQ121020, HQ397716, KR296812, EF641014, FJ435960, JX416368, KP099082, AY120903, AY120902, JX644908, AY324641, AY324638, AF159113.1, JQ413438, AF108861, GQ121021, KP730304, AY737602, AY731235, JX294358, AY268584, KR819168, AY120904.1, KU608308, AF112576, EU553551, JF710247, KP004205, JQ002555, KT235702, AB909498.1, AF316630, AY504515, AY504516, AF262325, GQ227479, EU827297, AF093500, AF093497, AY954886, HM116385, DQ650344, KY490554, KC305650, EF547155, FJ769050, HM243547, and AY524773 accession codes. The *gp60* sequence data that supported the CryptoCap_100k bait design were from GenBank with the FJ490060, FJ490087, FJ490058, JX412915, JX412926, JX412925, KC204983, and KC204984 accession codes. The twelve *Cryptosporidium* genome sequences used for CryptoCap_100k bait set characterization and *in silico* simulations were from GenBank with the GCF_00000165345.1, GCA_004337835.1, GCA_004337795.1, GCA_007210665.1, GCF_001865345.1, GCA_001865355.1, GCA_001593455.1, GCF_000006515.1, GCA_000165345.1, GCA_001483515.1, and GCA_002223825.1 accession codes *C. hominis* UdeA01 and *C. parvum* IOWA-ATCC were removed for those analyses with only ten genome sequences reference database (10-CrypGS). Generated fastq files from all *in silico* and *in vitro* experiments for mock and clinical samples are available in GenBank BioProject number PRJNA1061798.
